# Indigenous Values and Health Systems Stewardship in Circumpolar Countries

**DOI:** 10.3390/ijerph14121462

**Published:** 2017-11-27

**Authors:** Susan Chatwood, Francois Paulette, G. Ross Baker, Astrid M. A. Eriksen, Ketil Lenert Hansen, Heidi Eriksen, Vanessa Hiratsuka, Josée Lavoie, Wendy Lou, Ian Mauro, James Orbinski, Nathalie Pambrun, Hanna Retallack, Adalsteinn Brown

**Affiliations:** 1 Institute for Circumpolar Health Research, Yellowknife, NT X1A 3X7, Canada; hretallack@gmail.com; 2 School of Public Health, University of Alberta, Edmonton, AB T6G 1C9, Canada; 3 Institute of Health Policy Management and Evaluation, University of Toronto, Toronto, ON M5T 3M6, Canada; ross.baker@utoronto.ca (G.R.B.); adalsteinn.brown@utoronto.ca (A.B.); 4 Elders Council, Dene Nation, Northwest Territories, Yellowknife, NT X1A 1S1, Canada; francois.paulette@yahoo.ca; 5 Department of Community Medicine, The Artic University of Norway, 1909 Tromsø, Norway; astrid.eriksen@medisin.uio.no; 6 Regional Centre for Child and Youth Mental Health and Child Welfare (RKBU North), Faculty of Health Sciences, UiT the Arctic University of Norway, 9037 Tromsø, Norway; ketil.lenert.hansen@uit.no; 7 Health Care Centre, Municipality of Utsjoki, 99981 Utsjoki, Finland; heidi.eriksen@utsjoki.fi; 8 Southcentral Foundation, Anchorage, AK 99508, USA; VHiratsuka@SouthcentralFoundation.com; 9 Manitoba First Nations—Centre for Aboriginal Health Research, Winnipeg, MB R3T 2N2, Canada; Josee.Lavoie@umanitoba.ca; 10 Community Health Sciences, Faculty of Medicine, University of Manitoba, Winnipeg, MB R3T 0W3, Canada; 11 Dalla Lana School of Public Health, University of Toronto, Toronto, ON M5T3M7, Canada; wendy.lou@utoronto.ca (W.L.); orbinski@yorku.ca (J.O.); 12 Department of Geography, University of Winnipeg, Winnipeg, MB R3B 2E9, Canada; i.mauro@uwinnipeg.ca; 13 Dahdaleh Institute for Global Health Research, York University, Toronto, ON M3J 1P3, Canada; 14 National Aboriginal Council of Midwives, Montreal, QC H8R 3R9, Canada; nathaliepambrun@gmail.com

**Keywords:** health systems, values, indigenous, circumpolar comparison, stewardship, holistic approaches

## Abstract

Circumpolar regions, and the nations within which they reside, have recently gained international attention because of shared and pressing public policy issues such as climate change, resource development, endangered wildlife and sovereignty disputes. In a call for national and circumpolar action on shared areas of concern, the Arctic states health ministers recently met and signed a declaration that identified shared priorities for international cooperation. Among the areas for collaboration raised, the declaration highlighted the importance of enhancing intercultural understanding, promoting culturally appropriate health care delivery and strengthening circumpolar collaboration in culturally appropriate health care delivery. This paper responds to the opportunity for further study to fully understand indigenous values and contexts, and presents these as they may apply to a framework that will support international comparisons and systems improvements within circumpolar regions. We explored the value base of indigenous peoples and provide considerations on how these values might interface with national values, health systems values and value bases between indigenous nations particularly in the context of health system policy-making that is inevitably shared between indigenous communities and jurisdictional or federal governments. Through a mixed methods nominal consensus process, nine values were identified and described: humanity, cultural responsiveness, teaching, nourishment, community voice, kinship, respect, holism and empowerment.

## 1. Introduction

Circumpolar regions, and the nations within which they reside, have recently gained international attention because of shared and pressing public policy issues such as climate change, resource development, endangered wildlife and sovereignty disputes [1]. In response to these shared challenges, circumpolar nations have developed national-level strategies and related policies, which in turn drive objectives for foreign policy [2,3,4,5]. It has been stated that the interrelated elements of these policies have been instrumental in the construction of a new geopolitical space and a new, more inclusive circumpolar discourse [6]. Most prominent in this discourse are the Arctic states. The Arctic Council (a high-level intergovernmental forum) defines Arctic states as being inclusive of the United States of America, Canada, Iceland, Norway, Sweden, Finland, the Kingdom of Denmark (with the self-governing territories of Greenland and Faroe Islands) and the Federation of Russia. The Arctic council also recognizes with special status indigenous groups, and include representation of Sámi, Inuit, and First Nations through international organizations such as the Arctic Athabaskan Council, Aleut International Association, Gwich’in Council International, Inuit Circumpolar Council, Russian Association of Indigenous People of the North and the Sámi Council.

This geopolitical space is part of the broader aspects of the global health context [7]. Shared health challenges have been raised through various circumpolar forums for decades [8,9]. In these forums, there has been a significant focus on health disparities of indigenous peoples and the impacts of an intertwined range of health determinants such as food security, climate change and, in recent years, health systems [10,11,12,13]. In a call for national and circumpolar action on shared areas of concern, the Arctic states health ministers recently met and signed a declaration that identified shared priorities for international cooperation [14]. Among the areas for collaboration raised, the declaration highlighted the importance of enhancing intercultural understanding, promoting culturally appropriate health care delivery and strengthening circumpolar collaboration in culturally appropriate health care delivery. Reference to the health strategies such as the Kitigaaryuit Declaration endorsed by the Inuit Circumpolar Council emphasizes the need for collective approaches to address the health issues that arise across international boundaries in circumpolar regions in a way that reflects and respects indigenous values [15]. 

It is evident that the alignment of actors who influence health is complex and is further influenced as indigenous peoples’ transition from impacts of policies of assimilation, to post-colonial phases of governance and resulting redistribution of powers, in forms of decentralization and devolution of powers to regions, indigenous settlements and land claims. Engagement of local sectors is recognized to be of value and is quite prevalent in circumpolar regions. This type of engagement introduces another layer of governance that is not always equal to full devolution or decentralization, but is governance under a variety of agreements with varying levels of accountability. Despite the complexities of organization, there is a need to identify the common themes and context for circumpolar comparisons.

With these needs in mind, the authors have previously described how we might address health systems challenges in circumpolar regions, as well as highlighted the need to better understand the shared values and contexts [16]. The workshop findings included early indications that shared values and contexts exist between circumpolar regions. These values were seen to be rooted in indigenous traditions that are holistic and value contributions of the broader society. Although the importance of indigenous values is often discussed, such values have not been explicitly documented or explored in a circumpolar context as they may apply to frameworks that capture stewardship functions and performance measures.

This paper responds to the opportunity for further study to fully understand indigenous values and contexts, and presents these as they may apply to a framework that will support international comparisons and systems improvements within circumpolar regions. Specifically, we will explore the value base of indigenous peoples and provide considerations on how these values might interface with national values, health systems values and value bases between indigenous nations particularly in the context of health system policy-making that is inevitably shared between indigenous communities and jurisdictional or federal governments. 

### 1.1. Historical Background

Circumpolar nations share many experiences with the colonization of indigenous peoples and national policies of assimilation. This has had a twofold impact on indigenous peoples. Firstly, the indigenous health systems and traditions in place during the era of colonization were among the traditional institutions and activities that were suppressed and assimilated during colonial times. Secondly, these government policies (in some cases, health policies) have had devastating impacts on both the physical and mental health of indigenous people. The Romanow report on the future of Canada’s health care system highlights that “the health system must reflect the values, needs and expectations of all Canadians, including Canada’s Aboriginal peoples. The poor health status of Canada’s Aboriginal peoples is a well-known fact and a serious concern not only to Aboriginal peoples but also to all Canadians. The situation is simply unacceptable and must be addressed” [17]. It is not surprising to see that indigenous people’s satisfaction with, as well as their cultural relevance to, health care systems is poor in all circumpolar regions [18,19]. Since the 1970s, however, a policy shift has been evident in circumpolar countries, with indigenous groups taking on constitutional or legislative affirmations of their distinct status. This is demonstrated through national adoption of policies related to land rights, self-government, the upholding of treaties, the recognition of cultural rights and customary law, the guarantee of representation in central government, the constitutional or legislative affirmation of distinct status and the support or ratification of indigenous rights and affirmative action through international instruments [20]. Recent examples of circumpolar governments’ responsiveness include actions such as the establishment of the White House Council on Native American Affairs, an executive order that recognizes the inherent sovereignty and right to self-determination of indigenous nations [21]. A climate of acknowledgement of wrongs previously committed is exhibited through national apologies such as that of King Harald V of Norway, who expressed regret on behalf of the state for the injustice committed against the Sámi people through the harsh policy of Norwegianization, and the Canadian prime minister Stephen Harper’s apology on behalf of the Canadian government for harms caused by residential schools [22,23].

Specific to health, declarations such as the United Nations Declaration on the Rights of Indigenous Peoples have recognized the rights of indigenous peoples “to maintain and have access to their traditional medicines and health practices, including the conservation of their vital medicinal plants, animals and minerals”. The Declaration also calls for the “right to access, without any discrimination, all social and health services” [24]. However, while circumpolar nations have agreed to the terms of these declarations [24,25], there remains a lack of progress from health systems perspectives, including a lack of practical directive and understanding in improving and measuring systems-performance for indigenous peoples.

As we move forward, the intent of these agreements should be reflected in how we create health policy. In this climate of reconciliation, processes of governance and policy-making require a more comprehensive inclusion of indigenous values and deeper understandings of how these align with national values, as well as, ultimately, a collective approach that influences good stewardship and related policy. 

### 1.2. On Values and Stewardship

Use of the term “value” is widespread; it is not clear, however, what exactly values are and how they influence decision-making and good stewardship. In general, values have been referred to as a set of “relatively stable cultural propositions about what is deemed to be good or bad by a society” [26]. Theodore Marmor, Kieke Okma, and Stephen Latham describe values as individuals’ subjective views about what is worthy or important. Furthermore, they describe the forms values may take in considerations of health policy options. They highlight how, in a political context, statements of values may inspire, unite or even “constitute” a people, such as the case of the Declaration of Independence and the Bill of Rights in the United States. In other instances, “values of the common law or the values of the Catholic church, for example, are used to locate fundamental doctrines that emerge from the writings of, or the beliefs of the elite within, a certain tradition” [27].

There are many ways that values may interact, and one critical one—because of how it shapes the roles and scope of government activity, views of performance and the policy function generally—is stewardship. Stewardship is values-based and has been described as the “careful and responsible management of the well-being of the population”, and as the “very essence of good government” [28]. The World Health Organization (WHO) has highlighted stewardship as one of the four main functions of the health system (along with financing, creating and managing resources, and service delivery) [29]. A systematic review of the literature yields six generic functions of stewardship: strategy formulation and policy development, intersectional collaboration and action, health system governance and accountability, attention to system design, health system regulation and intelligence (data and analysis) generation [30].

A values-based approach to health systems stewardship in a circumpolar context, with its multitude of actors, requires a framework that is action-oriented and descriptive of fundamental systems elements that underlie systems control, directives and, ultimately, performance. Health systems stewardship is an approach that encourages decision-making that is ethical, fair and economically efficient. Stewardship requires a well aligned and consistent strategic direction [31]. It embeds health systems in wider society and takes into account not just government, but also all the actors who influence health, including the private sector and civil society [32]. 

Few frameworks exist that are underpinned by values that encompass comprehensive and respectful approaches that serve both indigenous groups and nations as a whole. In the context of national movements to improve system responses for indigenous people, and the need to repatriate indigenous ownership, a stewardship-based approach provides us with the opportunity to reflect on indigenous values and advance national goals to improve the efficiency and responsiveness of health systems.

In this paper, we explore the values of circumpolar nations and indigenous people. First, we reviewed national acts and multinational forums representative of four circumpolar nations (United States, Canada, Norway and Finland); and, secondly, we used a mixed methods consensus process to identify indigenous values in these nations.

### 1.3. Nordic and North American Values: Finland, Norway, the United States and Canada

At the national level, values serve as an important baseline that is visited and analyzed in assessing national tolerance for health reforms and advancement of innovations in policy frameworks. In circumpolar nations, the values that underlie health systems on the whole are highlighted to varying extents in ministry documents, and these values are often on the forefront of national debates on health care reform as governments aim to set priorities and respond to economic and contextual pressures. Values are being tested, for example, in the American discussion on the Affordable Care Act (ACA) [33]. 

National values, and the debates that surround them, are sometimes reflected in multi-national forums, white papers or national commissions. Table 1 lists a number of values as they have been highlighted in the four circumpolar nations examined in this study—Norway, Finland, the United States and Canada. Where our interest was in values, we differentiated stated values that were in fact goals. To capture a representation of national values underlying health systems, values were captured as described most recently in national acts and multi-national and national forums. These forums provide opportunities to reflect on these values and gauge the potential directions for good stewardship and related measures. Whereas the government documents merely state what the current values are, the reflective documents captured in larger forums provide some insight as to what should be an ideal or goal for a nation or a group of nations. 

The values underlying health systems have been reaffirmed through ministry forums such at the Economic Union [34], while national values have also been revisited through white papers [35] and national commissions such as the Romanow commission in Canada [17]. Most recently, activities have included the development of a new European policy for health, Health 2020, which is heavily influenced by the values and actions of the Nordic countries. As such, the document provides a unifying and overarching value-based framework for health development for countries with shared expectations based on shared values [36]. Overall the values that were captured in ministry documents were in fact goals that represented undefined values. For the purposes of focusing on values, these two aspects were differentiated in the table.

Within multi-national forums such as the WHO and the European Union (EU), not only does the identification of shared values help gauge tolerance for reforms, but shared values between nations can also foster collaboration and shared approaches. In the Tallinn Charter, for instance, the EU member states resolved to “promote shared values of solidarity, equity and participation through health policies, resource allocation and other actions, ensuring due attention is paid to the needs of the poor and other vulnerable groups” [37]. The health ministers of the 25 member states of the EU also called on European institutes to protect the values and principles that underpin the health systems of the EU as reconciling individual needs with financial pressures; the main feature of these systems is to make them financially sustainable in a way which safeguards these values into the future. As evidenced in Table 1, the overarching values of universality, access to good quality care, equity and solidarity have been widely accepted in the work of many EU institutions [34]. There remain significant differences, however, between Norway, Finland, the United States and Canada with respect to national values.

Section 19 of the Finnish Constitution guarantees the right to receive indispensable subsistence and care for all who cannot obtain for themselves the means necessary for a life of dignity. It states that the government must guarantee adequate social and health care services for all. Government responsibilities are also stipulated in the Finnish Local Government Act, the Primary Health Care Act, the Act on Specialized Medical Care and the Act on the Status and Rights of Patients. A number of international conventions, as well as the European Social Charter, also emphasize Finnish society’s responsibilities towards its members [42]. While the importance of health care in the event of illness is recognized, much greater significance is placed on sectors who influence health promotion and disease-prevention. To a very great extent, it is recognized that health is influenced by what goes on outside the health care system [43].

According to the National Health Plan for Norway (2007–2010), the government aims to strengthen and coordinate its focus on a more equal and fair distribution of good health. The principal goal is to prevent illness and harm. It is recognized that this does not involve only the health service, but also makes demands of all sectors of society that affect public health. The aim is for services to be of a high quality, and to be available within acceptable wait times and distances, reaching out to everyone regardless of their financial situation, social status, age, gender and ethnic background [40].

In the United States, however, there is greater support for market competition and entrepreneurship. Individual rights and personal responsibility play an important role in the United States’ political values. In recent years, the health care reforms within the ACA have brought debates about American values to the forefront. When fully implemented, the insurance reforms are expected to lead to coverage of 94% of the population [38]. Consisting of 10 separate legislative titles, the ACA has several major aims which demonstrate a shift in values (seen as an infringement by non-supporters). The first and most central aim is “to achieve near-universal coverage and to do so through shared responsibility among government, individuals and employers”. A second aim is “to improve the fairness, quality and affordability of health insurance coverage”. A third aim is “to improve health care value, quality and efficiency, while reducing wasteful spending and making the health care system more accountable for a diverse patient population”. A fourth aim is “to strengthen primary health care access while bringing about long-term changes in the availability of primary and preventive health care”. The fifth and final aim is “to make strategic investments in the public’s health, through both an expansion of clinical preventive care and community investments” [38]. 

Thomas Murray, in a commentary reflecting on American values inherent in the ACA reforms, highlights the broad range of values that Americans want the health care system to embody and pursue: not just liberty (which underlies the premise of choice for health care), but also justice and fairness, responsibility, medical progress, privacy and physician integrity, among others [33]. While the ACA primarily directs activities in such a way that individual liberties are maximized, there are also system approaches that are not as tightly linked to dominant American value orientations, such as programs established through Medicare, Medicaid, the Veterans Administration health program, the Indian Health Service (IHS), law mandating emergency medical care and tax incentives [27].

In the Romanow report on the future of health care in Canada, on the other hand, it is emphasized that Canadian values for health care are closely tied to understandings of citizenship, not privilege, status or wealth [17]. The principles for health systems articulated in the Hall commission report of 1964 [44] and the Canada Health Act of 1984 include public administration, comprehensiveness, universality, portability and accessibility. These five criteria have gained widespread public support in Canada [45]. With access to health services seen as a Canadian value in itself, by extension, the principles underlying the health system are often described to be values for health. 

Broader connotations of values for health have been captured in other forums. In 1997, the Values Working Group of the National Forum on Health explored the connections between Canadians’ core values and the health care system [26]. They identified several core themes that the public continues to support, including equity (of health and access), compassion, dignity and respect, efficiency/effectiveness, collective responsibility, personal responsibility, quality, thriftiness, responsible stewardship and accountability. The Canadian Health Services Research Foundation undertook an environmental scan to explore the shared values and principles, goals and key health policy issues across provinces and territories. They found jurisdictions are aiming to achieve health care that is person-centered, accountable, efficient and equitable [46].

In Canada, we see higher levels of governmental control over the health system, and the United States maintaining elements of individual choice. Overall, from a governmental perspective, values underlying health systems in Canada and the United States are more operational and oriented to the “health system” in itself, versus values for wellness that influence actions in sectors outside health. Values described in Norway and Finland, however, are more oriented to values underlying a broader connotation of health and wellness for individuals and society, resulting in a process being more oriented to health systems stewardship and as a result, being more encompassing to health policy moving across sectors. 

While some forums consider indigenous values not being reflected within current health systems, there is little published work that identifies the specific values expressed by indigenous people. The following section describes the consensus mixed methods process by which we examined/identified the indigenous value base, followed by a more detailed description of the values themselves.

### 1.4. Exploring Indigenous Values

#### Objective

The objective of this study was to explore and describe the indigenous values that underlie and direct effective health systems stewardship in circumpolar countries including the United States, Canada, Finland, and Norway.

## 2. Materials and Methods

We explored the values underlying health systems stewardship through a collaborative consensus-based approach with indigenous scholars and knowledge holders. This methodology is described in detail elsewhere and explains the study elements in more detail [47]. We used a mixed method approach with indigenous knowledge and a nominal group process. Nominal group processes were originally designed to capture qualitative information for health planning. The process allows for engagement of all participants in the development of the question and process [48]. This workshop was based at a fly in lodge in northern Canada. The setting was a deliberate selection that was would allow for the expression of traditional knowledge and accommodate gatherings within the consensus process. An embedded, transformative, emergent mixed methods design was used in this study [49]. An embedded design entails the collection of one type of data (traditional knowledge) within a design framework associated with another type of data (nominal group process). As such, this embedded approach included indigenous knowledge within a study design that is more familiar to management sciences. A transformative approach ensures that the study is adaptable, respectful, and responsive to indigenous knowledge [49]. As such, the process was iterative and the resulting consensus based mixed methods approach included both Western and indigenous knowledge, striving to bridge the gap between health systems scholarship and indigenous scholarship and inform representative findings. 

While the first author of this paper (Susan Chatwood) designed the study, provided overall organization and facilitated the consensus methods, the remaining authors contributed to the design through the iterative process, through contributions in embedding the participatory data and in the analysis of findings. (Francois Paulette) co-facilitated and provided leadership in matters related to indigenous knowledge and facilitated matters related to local protocol and ceremony. Acknowledging that narrative approaches are more conducive to capturing some aspects of indigenous knowledge, team members with expertise in transferring traditional knowledge through media prepared a film on the workshop that captured some elements of the findings and experiences of the participants [50]. This mixed approach to dissemination is seen to be of value in reaching a number of stakeholders. 

### 2.1. Participants

A heterogeneous group of ten experts from the circumpolar regions of the United States, Canada, Norway and Finland were brought together. Participants identified as First Nations, Inuit, Métis, Sámi and non-indigenous. While English was the first language of only four participants, it was the common language of the group and used for the majority of the workshop. Other languages spoken included Chipewyan (Denesuline), Sámi, Norwegian, Finnish, and French. Participants had varied and combined backgrounds that included experiences as researchers, health care professionals, informal caregivers, indigenous leaders, elders, health managers and clinicians. The research experiences and knowledge bases of the experts were in the areas of health systems, health status in circumpolar countries and indigenous knowledge, as well as in mixed methods in an academic and indigenous knowledge context. Criteria for inclusion included experience in health systems operations, lived experience in indigenous and circumpolar contexts and traditional and academic models of research and knowledge. Participants were first selected through circumpolar networks, then referrals were made and participants were gathered until there was representation of indigenous groups and health systems perspectives (research, clinical, policy, and indigenous knowledge). 

The small number of participants (n = 10) was deliberate and is common in nominal group methods where the aim is to attain a high level of engagement and dialogue [51]. In addition, due to small populations and the nature of this subject specialty, there are generally smaller numbers of subject area experts in circumpolar health research. 

### 2.2. Process 

**Figure 1:**
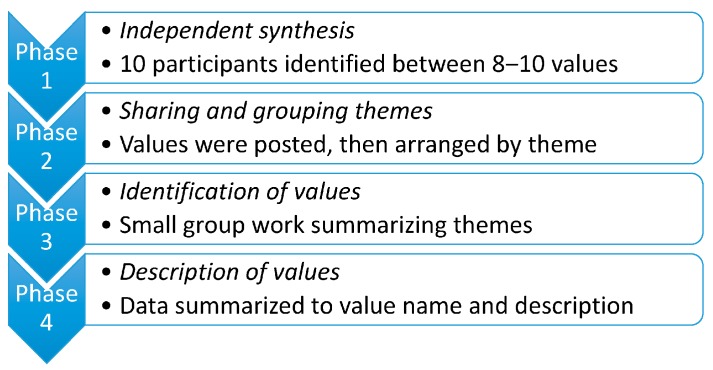
Four phases of the consensus process

Figure 1 highlights the four phases of the consensus process, followed by a more detailed description of each phase. The details of this process are described by the authors elsewhere [47]. The data detail for each value described can be found in Table A1.

Phase 1: At the beginning of the process, participants were asked to work independently and to write values on out cards provided. This component was done independently to maintain an anonymous process and to allow each participant to express his or her views without influence. 

Phase 2: Each participant put forward six of their selected values and the group’s combined chosen values were placed on a wall for all to view. A facilitated and interactive process with discussion between participants allowed for values to be grouped, and discussion around the themes and allocation of values took place in groups. 

During the breaks from the hands-on process, participants shared more indigenous based knowledge via film, stories and ceremony, which contributed to deeper understandings of values, and created opportunities for sharing within a multicultural group. 

Phase 3: The third phase entailed assigning a description to the value groupings. Upon completion of the individual or small group work, information was shared in a large group session where the descriptions were discussed. Consensus was further built through this process. At this point, nine themes were identified. 

Phase 4: Each value description shared in the face-to-face session was recorded on a spreadsheet and put in a shared on-line workspace for all participants to view. The value descriptions were then summarized through written feedback and telephone conversations, and, finally, a heading was assigned to each value. This component was carried out by email collaboratively after the face-to-face workshop. 

## 3. Results

Through these mixed methods participatory process of consensus-building, nine values were identified and described: humanity, cultural responsiveness, teaching, nourishment, community voice, kinship, respect, holism and empowerment. The values were left intentionally broad with the understanding that they overlap and interact with one another. 

During the workshop, we heard stories related to specific program applications that were reflective of indigenous values. The examples highlighted provide some perspective on how system responses may play out on the ground. Again, it should be emphasized that the values are interconnected, and the provided examples of each can be relevant to more than one value. These examples are provided, however, for the purpose of highlighting the value being described in a health system context. 

### 3.1. Humanity

The value of humanity emphasizes the fundamentality of relationships between human beings. It also recognizes the aspects of those relationships, including empathy, sensitivity, respect and care, that sustain a wholesome life, build trust and bridge conflict in cross-cultural settings. 

Examples highlighted by the group included the people-centered care models that are being developed by indigenous people, such as the Nuka System of Care in Anchorage, Alaska, which is built on healthy relationships. The vision and mission focus “on physical, mental, emotional, and spiritual wellness and working together as a community” [52]. 

### 3.2. Cultural Responsiveness

The value of cultural responsiveness encourages processes and protocols that focus health care on community values and culture, drawing on indigenous/traditional knowledge, languages and styles of communication. 

In applications, this could be seen to encompass the engagement of indigenous knowledge via the engagement of indigenous peoples in all aspects of care. Instances where this has been applied include the case of reserved seats for Sámi in medical education in Tromso, Norway [53], the law requiring availability of Sámi interpreters and upholding language rights in Finland, a general law which has specific impacts for health [54]. 

### 3.3. Teaching

This value urges that traditional teachings have a central place in the education and training of caregivers and other people who work in health systems. It also supports cultural sensitivity by promoting a knowledge exchange among health-care workers, researchers, and communities that incorporates a holistic view of the interconnectedness of traditional spiritual and environmental laws and an understanding of the natural order.

A reference program that exemplifies this value is the midwifery education program in Nunavik at the Inuulisivik Health Centre [55]. This internationally recognized program includes the training of Inuit midwives within the community based birthing services program and is seen to be integral in fulfilling program goals to improve community health and nurture wellness. Other examples of this value include, the affirmative action policy that exists to recognize the representation of Aboriginal peoples through human resources policies and aims to enhance the competence of services in Canada’s Northwest Territories [56].

### 3.4. Nourishment

This value recognizes the importance of water and food as nourishment to achieve balanced health, emphasizes local/traditional food and the sharing of food and recognizes the need to use resources wisely and to ensure equitable access.

Some examples of this value in action include dietary protocols and policies for families to bring in outside food to be prepared at hospitals, and staff training for indigenous nutrition needs in Whitehorse, Canada [57]. The ability to access traditional foods while recovering from illness in a hospital setting is integral to healing and establishing balance.

### 3.5. Community Voice

Community voice urges that the traditional and contemporary values of the community drive the design, processes and delivery of health care. Community members’ shared histories, experiences, language(s), and economy/trades shape how we conceive of health, experience health care, develop trust in health care systems and interact with Western medical systems. Access to quality health care for all members of the community is crucial.

An example of the value of community voice in practice is the Elders’ Council and its mandate to inform hospital/health authority policy and ensure services are more responsive to indigenous families at the Stanton Territorial Health Authority in Yellowknife, Canada. Another example is Inuit Qaujimanituqangit (Inuit traditional knowledge) and Nunavut Government’s IQ framework in Nunavut, Canada which provides guidance on how traditional knowledge is included in policy and programs for the territory [56]. 

### 3.6. Kinship

This value prioritizes family as an expanded network of kinship associations. It maintains that family is sacred and gives a sense of place and where you come from, recognizing each person’s unique contribution to family in the context of home and the land.

The value of kinship is evident in the midwifery legislation that aims to recognize teaching and continuing education addressing Inuit culture in Puvirnituq, Nunavik [58].

Another example is the Southcentral Foundation facility design strategies of Anchorage, Alaska, which aim to accommodate family and community gathering through open spaces and accommodations through design to gather [59]. 

### 3.7. Respect

This value dictates the manner in which interpersonal and community-to-community interactions should take place—that is, with mutual respect for differences within and between families and communities, respect of traditions, traditional knowledge, and traditional healing methods and respect through active listening, trust, sensitivity, transparency and consensus.

This value is evident in Southcentral Foundation’s inclusion of traditional healers on an accredited medical center campus in Anchorage, Alaska. Another example is Canada’s Non-Insured Health Benefits transportation policy, which allows patients to access traditional healers—noting the policy has jurisdictional limitations related to portability of services when needing to travel outside province or territory. These programs strive for comprehensive services though respect of traditions. 

### 3.8. Holism 

This value involves having a holistic view of a person’s ties to land, home, traditions, values, distinctive roles and responsibilities and boundaries/possibilities. It recognizes one’s place in the continuity of space, time, location and purpose, and emphasizes interconnections between the quality of our mental, physical, emotional and spiritual lives.

A land-based camp for mental health services (*meahcceterapiija*) through Sámi National Centre for Mental Health (SANKS), in Karasjok, Norway, exemplifies this value by recognizing the importance of connections with the land and relationship this has to family and healing [60].

### 3.9. Empowerment

This value promotes the sense of worth and empowerment of individuals, families and communities that is derived from understanding one’s place in the natural order and one’s ties to land and tradition. It involves establishing community care based on the needs, ways of thinking, and holistic perspectives of indigenous peoples to preserve dignity and support. It stresses that informed decisions promote autonomy and independence. 

The messaging regarding the perspectives of the holism, relationality and interconnectedness of the values was strong and emphasized many times during the workshop. The lines drawn between the nine values are somewhat arbitrary, and are presented only to demonstrate the multiple levels and constructs represented. As such, the values cannot be separated, but are to be viewed as part of a whole:

“*When all that is put together—in my language simply we refer to this as “Dene Ch’anié” … It is descriptive of everything, our history, our spiritual, laws, environmental laws, political laws, economic laws, of how people are to live together, to interact. Protocols of living and families, communities and others. So for me, “Dene Ch’anié” is the best word I can use to describe this*”.

—workshop participant

The initial findings, then, capture the values that were shared across groups and demonstrate there are some commonalities in indigenous values underlying health systems stewardship in circumpolar regions within Sámi, Inuit, First Nations and Métis peoples. The detailed data from the workshop is included as a supplement to this article. Of course this does not discount the variation between and within cultural groups, but does provide support for commonalities that can support the refinement of stewardship functions through benchmarking and enhance collaboration and systems performance across circumpolar countries.

## 4. Discussion

As stated earlier, circumpolar nations have shared histories of national policies of assimilation and suppression of values and beliefs of indigenous people in those respective nations. Ultimately, this period and its resulting policies have had detrimental impacts on both health outcomes and traditional systems of indigenous peoples. Indigenous perspectives within health debates have been captured within the lens of equity, and as such indigenous needs are often framed as belonging to disadvantaged and marginalized populations, as opposed to more strengths-based systems that define people in nations. However, important shifts are occurring within nations. Coupled with increasing understanding of the intent of national treaties and autonomy of indigenous groups, these shifts create a more comprehensive representation of the national context and a positive environment for good health systems stewardship, resulting in policy frameworks which are built on shared and inclusive values. Overall, there is an emerging climate of reconciliation and cohesion that acknowledges indigenous and national values in a more complex, yet inclusive manner. In turn, this national dialogue can drive more respectful value bases that will inform health debates and policy frameworks for all residents of circumpolar nations. 

While the indigenous values underlying health systems have not been consistently described in the literature, the Romanow report on the future of Canadian health care makes special mention of the indigenous vision of health care, “in which each person is considered as a whole, with health and social problems that cannot be cured in isolation from one another, and with resources for achieving health that come not just from expert services but also from the understanding and strength of family, community, culture and spiritual beliefs. It is a vision quite different from that of mainstream health and social services, which tend to isolate problems and treat them separately” [61]. The report features a quotation by Henry Zoe, member of the legislative assembly in Northwest Territories, Canada, from December 1992, which provides a nice summary:

“*For a person to be healthy, [he or she] must be adequately fed, be educated, have access to medical facilities, have access to spiritual comfort, live in a warm and comfortable house with clean water and safe sewage disposal, be secure in their cultural identity, have an opportunity to excel in a meaningful endeavour, and so on. These are not separate needs; they are all aspects of a whole*”.

The workshop described above allowed us to follow a consensus process and hear stories reflecting indigenous knowledge related to specific program applications that were reflective of indigenous values. It is recognized that the values generated by this workshop are neither a final product nor one that is applicable in all sectors providing health services to populations with indigenous representation. Rather, this is seen as a starting point in recognizing the importance of indigenous values in national and circumpolar contexts for health systems stewardship. The examples highlighted provide some perspective on how system responses to indigenous values may play out “on the ground”. The linking of indigenous values with health systems stewardship frameworks aims to operationalize at a higher level how we might bring indigenous perspectives to the core of good stewardship and facilitate health directives as a component of national agendas to reform policies that previously repressed and assimilated indigenous peoples. The ultimate aim is to achieve better health outcomes for all. 

To this end, further consideration of the relationship between indigenous values and national values is required. The interface of indigenous values with overarching national values and consistency of stewardship is a complex interface of constructs; however, it is worthy of further study to guide us to enhanced stewardship in circumpolar nations. As noted earlier, Norway and Finland have value systems that are more holistic in nature, while Canada promotes values oriented to a more narrowly defined system, with the United States promoting value for the health system that is limited to activities within health sectors. Despite these differences, and in an attempt to elicit some discussion on the alignment of what are suggested as values underlying national health systems on one the hand, and indigenous values on the other hand, a preliminary table was developed outlining their similarities and differences (see Table 2). 

As is evident, many of the national values align well with the indigenous values as described in this exercise. It is noted that the majority of these values come from broader international documents that aim to encompass health systems in a broader stewardship-based model. One exception to this is the U.S. values around liberty, justice, and fairness, which align with the call for community voice and empowerment in some contexts. It could also be interpreted that liberty as it aligns with individual needs could conflict with community needs. The values that capture aspects of relationships via teaching, families (kinship) or ties to the land (nourishment) fell outside of the values described in national reports. However, given the interrelated aspects of the values, it cannot be said these lie outside the scope of other national values. 

The definition of values at the national level and their relationship to elements of stewardship including systems reforms, policy development and performance measurement is a complex and often debated topic [27,35,62]. For nations to reach a deeper acknowledgement of indigenous values within existing systems is an ongoing process. The reaffirmation of indigenous values informs a proactive values-based approach that is inherent in good stewardship and nationhood during these times of reconciliation. A well-articulated and mapped process can provide a mechanism to uphold stewardship functions that are values based, responsive, engaging across sectors and empowering to indigenous populations. The specific mechanism by which we may incorporate values into a health systems stewardship framework merits further study. 

It is worth emphasizing, again, that stewardship goes beyond government command and control models of governance, but is more holistic and inclusive across sectors. As indigenous values are captured in the conceptions of good stewardship and phases of implementation are advanced, key sectors to guide this process are those in which indigenous groups have high levels of autonomy and the ability to control and design the systems according to values and need. Common understandings of values can enhance communication between stewards, be they health departments in government, indigenous governments or community-recognized elders. 

The ability to articulate indigenous values as a foundation of good stewardship provides guidance for responsive and equitable strategies that enhance the ability of stewards to fulfill the following six generic stewardship functions: strategy formulation and policy development, intersectional collaboration and action, health system governance and accountability, attention to system design, health system regulation and intelligence (data and analysis) generation [30]. While the workshop participants highlighted some examples of systems practices that are responsive to indigenous values, there is a need for a more systematic study of indigenous values and how they align with specific stewardship functions within nations. 

## 5. Conclusions

In this paper, we have captured a representation of nine indigenous values that underlie health systems stewardship in circumpolar nations. As stated, these values are interconnected and have unique interpretations at the community level, and as such require ongoing consultation and interpretation. While nations represented in this study were limited to four of eight arctic states (the United States, Canada, Norway and Finland), there was a comprehensive representation of indigenous groups within circumpolar nations, including Inuit, First Nations, Sámi, Métis and non-indigenous. The findings of this initiative articulate a previously suppressed value perspective within national health systems due to policies of assimilation for indigenous peoples. The findings of this study introduce a process that may broaden the articulation of national values and provide a basis for further study and applications for good stewardship and international comparisons. 

Overall, the identification of indigenous values in informing ethical stewardship of health systems was seen in this study to be a positive, proactive and empowering approach that was built on trust and the strengths of indigenous nations. The commonality in values between countries highlights the potential for international collaborations and comparisons between countries, as nations move towards reconciliation, health systems improvements and improvements to the livelihood of indigenous peoples. While program elements in relation to values were described, there is an ongoing need to understand how indigenous values align with national values and stewardship functions, with an aim to improve health systems responsiveness and performance in an indigenous context, and advance national goals of improving efficiencies, population health and system responsiveness for all. 

## Figures and Tables

**Table 1:** Values described for health care in forums and acts.

Values or Goals That Represent Values	Health and Policy Forums				National Health Acts			
	Health 2020 [36] EU	Tallinn Charter WHO Europe [37]	Canada Romanow Proposed Health Covenant [17]	USA [33]	USA PPACA [38]	Canada Health Act [39]	Norway (National Health Care Services Plan) [40]	Finland Objectives (Health Care Act) [41]
Values								
Justice and Fairness				X	X			
Solidarity	X	X						
Dignity	X							
Non-discrimination	X							
Liberty				X				
Respectful			X					
Goals representing undefined values								
Universality	X		X		X	X		
Equity (access and outcomes)	X	X	X				X	
The right to participate in decision-making or * (mutual responsibility and public input)	X	X	X *			X		
Accountability Or *(democracy and legitimacy)	X		X		X		X *	
Access to care (responsiveness) ^#^	X		X ^#^			X	X	
Client-orientation or * (stronger patient role)			X				X *	X
Strengthen cooperation or * (cohesion and interaction) or ^#^ (expansion of clinical preventative care and community investments)					X ^#^		X *	X
Portability (proximity and security) *			X			X	X *	
Public Administration			X			X		
Promote health and welfare (work and health) *							X *	X
Efficiency and Effectiveness (professionalism and quality) *			X				X *	
Sustainability (value, quality, and efficiency) *	X				X *			
Comprehensiveness						X		
Transparency	X		X					
Medical progress				X				
Privacy				X				
Physician Integrity				X				
Reduce health inequalities								X
Ethical			X					
Strengthen primary care access and preventative care					X			

Within documents original language was retained and grouped *, ^#^ used to identify where language aligns across rows in table.

**Table 2:** Alignment of National and Indigenous Values.

Values Identified in National Documents	Indigenous Values Identified by Consensus Process
Dignity (Health 2020)/Ethics (Romanow report)	Humanity
Liberty (USA)/Solidarity (Health 2020, Tallinn)	Community voice
Justice and Fairness (of health care insurance) (USA)	Empowerment
Respect (Romanow report)	Respect
Non-discrimination (Health 2020)	Cultural responsiveness
-	Teaching
-	Nourishment
-	Kinship
-	Holism

## Supplementary Materials

Supplementary File 1

## Appendix A

Detailed data on values from consensus process.

**Table A1:** Data from consensus process.

Phase	Values								
	Teachings	Cultural Responsiveness	Respect	Community Voice	Holism	Kinship	Empowerment	Nourishment	Humanity
**Phase 1 & 2**									
**Values from Independent synthesis**	Learn and do what you lean = teach Education and training Indigenous context in the education system Capacity building Traditions	Language and communication Indigenous knowledge/understanding Knowledge All things are connected Traditions Language Protocols Communication Traditional Knowledge	Understanding respect for different world view(s) Respect Reciprocity Reciprocity Honor the elders Honour Respect to culture Show respect to others Sacred	Boundaries Empowerment Resurgence of traditional values + way Sovereignty Place/land History Life Traditional Knowledge decolonization ties to homeland Time vs. timelessness	“Circle” Holistic Holistic perspectives Diversity Diversity interconnections	family family know who you come from Home, love and respect to land	short distance to hospital/health care co-ordinate for the patient not the system patient in centrum “situated response” process that respects culture/spirit and relations availability lifespan perspective access community driven	Food/sharing nourishment water housing traditional medicines livelihood environment connection	care acceptance sympathy/empathy have patience together emphathy see the humor/laugh/smile compassion pray for guidance listening love take care of others caring responsibility live carefully patience respect share what you have sensitivity accept life as it occurs
**Phase 3**									
**Group work summariaing themes**									
**Group 1**	Collaboration through indigenous knowledge transmission and knowledge receipt to achieve continuity in health and/or shared outcomes	Processes that must be reconceived to conduct community values centered health care	Manner in which interpersonal and community to community interactions should take place in	Historical legacies influence community conception of health, power relationships in health systems, and recognizes/leverages indigenous knowledge and the significance of forbearance in indigenous culture(s)	Recognition of place in the continuity of the cosmos, including space, time, place and purpose. Includes conception of distinctive roles, responsibilities, and restrictions/possibilities.	Community members’ shared histories, experiences, language(s), economy/trades which shape how we conceive health, experience health care, develop trust in healthcare systems, and interact with western medical systems.	“Community driven health care” The traditional and contemporary values of the community (ies) drive design, processes, and delivery of health care.	Structural and systematic influences on health and wellbeing with locus of control varying due to sociopolitical climate	Self-regulation concepts/Series of beliefs (mindset, spirituality) conducive to or complementary to a wholesome/full life
**Group 2**	High quality culture sensitive health care services in own langauge		Respect of traditions, traditional knowledge and traditional healing methods	Established (by indigenous peoples) community care based on the needs, way of thinking, holistic perspective, of the indigenous peoples (instead of “translating” the systems of majorities to an indigenous language)	“circle” biopsychosocial			FOOD, local traditional food and nourishment	
**Group 3**	shared research, self-reflection for person doing the work, collaboration, cooperativeness		+Active listening +trust. Respect embraces sensitivity and transparency and consensus	Preserving dignity, responsible, informed decisions that promote autonomy and independence	Maintenance of quality of our mental physical emotional spiritual life; we‘re in our highest functioning way			Sustainability, wise use of resources, equity in distribution and access to those resources	Humanitarian way of doing things; this value is foundational to many of the other ways
**Group 4**	Traditional teachings have a central place in education and training of caregivers and health authorities; must look at spiritual and environment Laws → natural order Teaching must have a holistic view which must be shared with people who work in the field of health Interconnectedness must be part of the health system, health policy, and health directives	Protocols and clear communication need to be addressed, whether the protocols come from traditional knowledge or from language	Mutual respect (“two-way street”)	See holism	When people have a stable place they know their history tied to land and home, sense of tradition and values. They understand their place on the land. They understand their boundaries [physical boundaries tied to land] à empowerment [being rooted in own land and understanding how others fit in]. People with a sense of worth. Having this knowledge can lead to decolonization. Sense of empowerment and sovereignty can then emerge [ß has led into OURS]	Home, respect for the land. Family must know who you are related to, sense of extended family, must know where they came from and their place in the family. Everyone has a gift that they contribute to their family (unique contribution). The whole family must know their identity.Important to see family as sacred, see sacredness of families as en entity. Worldview must be reflective and respect differences within a family, between families, within and between communities	Services must be available, all must have access to hospitals and health centers. People in communities must have say in what services are provided at the community level	Water is essential to the health of people, whether living on water or land. Nourishment, food security, sharing of food;—all must have their place in hospitals and health authorities for people to access in order to maintain balanced health	humans struggle to strive for peace; conflict has always been a problem Often in cross-cultural situations need time to build trust—for wellbeing of both parties
	**(emphasising interdepence) When everything is said and done, it’s all about diverse communities and their interdependence**								
**Phase 4**									
**Description of values**	Traditional teachings have a central place in the education and training of caregivers and other people who work in health systems. Supporting cultural sensitivity by promoting a knowledge exchange among healthcare workers, researchers, and communities that incorporates a holistic view of the interconnectedness of traditional, spiritual, and environmental laws and an understanding of the natural order.	Having processes and protocols in place that focus healthcare on community values and culture, drawing on Indigenous/traditional knowledge, local languages, and styles of communication.	The manner in which interpersonal and community-to-community interactions should take place. Mutual respect for differences within and between families and communities. Respect of traditions, traditional knowledge, and traditional healing methods. Respect through active listening, trust, sensitivity, transparency, and consensus.	The traditional and contemporary values of the community drive the design, processes, and delivery of healthcare. Community members’ shared histories, experiences, language(s), and economy/trades shape how they conceive of health, experience healthcare, develop trust in healthcare systems, and interact with Western medical systems. Access to quality healthcare for all members of the community.	Having a holistic view of a person’s ties to land, home, traditions, values, distinctive roles and responsibilities, and boundaries/possibilities. Recognizing one’s place in the continuity of space, time, location, and purpose. Interconnections between the quality of our mental, physical, emotional, and spiritual lives.	Family as an expanded network of kinship associations. Family is sacred and gives a sense of place and origin. Recognizing each person’s unique contribution to family in the context of home and the land.	Promoting the sense of worth and empowerment of individuals, families, and communities as derived from understanding one’s place in the natural order and one’s ties to land and tradition. Establishing community care based on the needs, ways of thinking, and holistic perspectives of Indigenous peoples to preserve dignity and support. Informed decisions promote autonomy and independence.	Recognizing the importance of water and food as nourishment to achieve balanced health. Emphasizing local/traditional food and the sharing of food, and recognizing the need to use resources wisely and to ensure equitable access.	Emphasizing the fundamentality of relationships between human beings. Recognizing aspects of those relationships, including empathy, sensitivity, respect, and care, that sustain a wholesome life, build trust, and bridge conflict in cross-cultural settings.
